# Impact of early vs. late tracheostomy on clinical outcomes in mechanically ventilated patients with intracerebral hemorrhage extending into the ventricles: a retrospective cohort study based on quantitative assessment of parenchymal and intraventricular hematoma volumes

**DOI:** 10.3389/fneur.2026.1724717

**Published:** 2026-01-30

**Authors:** Minghui Lu, Jiajun Wei, Qiang Cai

**Affiliations:** Department of Neurosurgery, Renmin Hospital of Wuhan University, Wuhan, Hubei, China

**Keywords:** complications, critical care, intracerebral hemorrhage extending into the ventricles, mechanical ventilation, quantification of hematoma volume, risk factors, timing of surgery, tracheostomy

## Abstract

**Background:**

The optimal timing for tracheostomy in patients with intracerebral hemorrhage extending into the ventricles who require mechanical ventilation remains controversial, and there is a paucity of evidence to guide clinical practice. This study aimed to elucidate the impact of early vs. late tracheostomy on clinical outcomes and complications in this population, utilizing multivariable models to identify risk factors and define the potential beneficiary population.

**Methods:**

This single-center retrospective cohort study consecutively enrolled 157 patients with severe spontaneous intracerebral hemorrhage extending into the ventricles requiring mechanical ventilation (GCS score ≤8) between January 2020 and December 2023. Based on the timing of tracheostomy, patients were classified into an early group (ET, ≤7 days after mechanical ventilation, *n* = 81) and a late group (LT, >7 days after mechanical ventilation, *n* = 76). Baseline characteristics, treatment measures, and outcome data were collected. Hematoma volumes in both the brain parenchyma and ventricles on admission CT scans were precisely quantified using 3D Slicer software. The primary outcome was the 6-month modified Rankin Scale (mRS) score. Secondary outcomes included the duration of mechanical ventilation, ICU length of stay (LOS), and the incidence of short-term complications [ventilator-associated pneumonia (VAP), new-onset arrhythmia, shock, and acute kidney injury (AKI)]. Multivariable logistic regression analysis was employed to identify independent risk factors for complications and to assess the protective effect of early tracheostomy.

**Results:**

In this cohort of 157 mechanically ventilated patients with severe intraventricular hemorrhage, baseline characteristics were well-balanced between Early (ET, *n* = 81) and Late Tracheostomy (LT, *n* = 76) groups. While 6-month functional outcomes (mRS) showed no significant difference (*P* = 0.360), the ET group demonstrated substantially shorter duration of mechanical ventilation (13 vs. 19 days, *P* < 0.001) and ICU stay (17 vs. 25 days, *P* < 0.001). ET was associated with significantly lower incidence of ventilator-associated pneumonia (28.40 vs. 48.68%, *P* = 0.009), new-onset arrhythmia (18.52 vs. 32.89%, *P* = 0.039), and shock requiring vasopressors (24.7 vs. 40.79%, *P* = 0.031). Multivariable analysis identified GCS score <6 (OR 3.588, *P* = 0.008) and Graeb score ≥8 (OR 8.735, *P* = 0.037) as independent risk factors for complications, while confirming early tracheostomy as an independent protective factor (aOR 0.306, *P* = 0.019) after adjustment for confounders.

**Conclusion:**

In this single-center retrospective cohort study, early tracheostomy was associated with shorter durations of mechanical ventilation and ICU stay, as well as a lower incidence of major complications, and demonstrates a favorable safety profile. Although it does not improve long-term neurological function, early tracheostomy serves as an independent protective factor. When combined with the identification of risk factors such as GCS <6 and Graeb score ≥8, it provides a basis for individualized treatment. These findings suggest an association that warrants further investigation in prospective studies.

## 1 Introduction

Intracerebral hemorrhage extending into the ventricles represents one of the most devastating stroke subtypes, posing a significant global public health challenge due to its high rates of mortality and disability (1). The prognosis for these patients is exceedingly poor, primarily owing to a complex pathophysiology that includes primary parenchymal injury, acute hydrocephalus, chemical meningitis triggered by bloody cerebrospinal fluid, and sudden increases in intracranial pressure (2–4). Consequently, prolonged mechanical ventilation is often necessitated by depressed consciousness, impaired airway protective reflexes, and elevated intracranial pressure (5).

Prolonged mechanical ventilation is associated with significant complications, including ventilator-associated pneumonia (VAP) and laryngeal injury (6). Tracheostomy serves as a pivotal intervention to address this challenge, providing a secure airway, enhancing patient comfort, reducing sedation requirements, and facilitating ventilator weaning and rehabilitation (7). However, the optimal timing for this procedure in neurocritical patients, particularly those with intracerebral hemorrhage extending into the ventricles, remains a subject of ongoing debate (8).

Previous studies and systematic reviews in general critically ill populations suggest that early tracheostomy (typically ≤ 7 days) may reduce the duration of mechanical ventilation, the incidence of ventilator-associated pneumonia, and ICU length of stay (9–11). In contrast, a late strategy (>7 days) might avoid unnecessary procedures in some patients and circumvent risks associated with coagulopathy during the acute phase. However, whether these findings are generalizable to the intracerebral hemorrhage (ICH) population requires further validation. Patients with ICH extending into the ventricles possess a distinct injury mechanism and pathophysiological profile. There is a scarcity of research focusing on tracheostomy timing in this specific cohort. Furthermore, the timing decision should not rely solely on a universal time threshold but ought to be individualized based on the objective severity of the primary brain injury (12).

Tracheostomy is essential for ICH/IVH patients requiring prolonged ventilation, though it carries risks such as bleeding and infection. The optimal timing of the procedure remains unclear. Our analysis systematically incorporates quantified parenchymal and intraventricular hematoma volumes, and employs multivariable models to adjust for prognostic factors and identify outcome associations. This study examines the relationship between tracheostomy timing [early ( ≤ 7 days) vs. late (>7 days)] and clinical outcomes in severe ICH/IVH. We hypothesize that early tracheostomy is associated with fewer ventilator-associated complications and shorter ICU stays, but not with improved long-term functional outcomes.

## 2 Methods

### 2.1 Study design and ethical approval

This was a single-center, retrospective, observational cohort study. Ethical approval was obtained from the Ethics Committee of Renmin Hospital of Wuhan University (Approval No. WDRY2022-KS002). Due to the retrospective nature of the study using anonymized data from routine clinical practice, a waiver of written informed consent was granted.

### 2.2 Patient population

**Figure 1:**
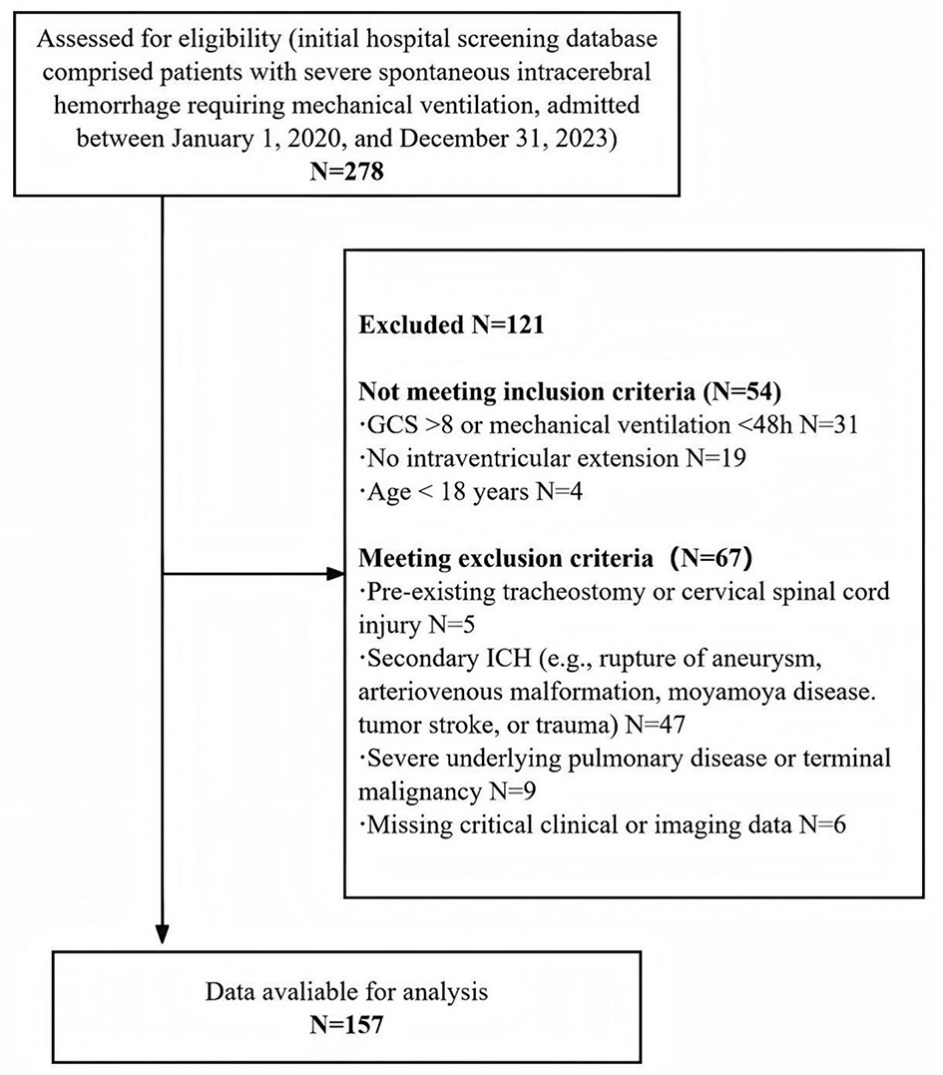
Flow chart for patient selection. Information on 278 patients was collected, of which 121 patients were excluded due to multiple reasons, and finally 157 patients were included for further analysis.

This study involved a retrospective analysis of mechanically ventilated patients with severe spontaneous intracerebral hemorrhage at Renmin Hospital of Wuhan University between January 1, 2020 and December 31, 2023. The inclusion criteria were: (1) age ≥18 years; (2) first-ever spontaneous supratentorial intracerebral hemorrhage with radiologically confirmed extension into the ventricular system, as independently verified by two neurosurgeons; (3) Glasgow Coma Scale score ≤ 8 on admission and mechanical ventilation duration ≥48 h; and (4) tracheostomy performed during hospitalization. Patients were excluded for any of the following: (1) secondary intracerebral hemorrhage due to aneurysm, vascular malformation, tumor, or trauma; (2) pre-existing tracheostomy or cervical spinal cord injury; (3) severe underlying pulmonary disease or advanced malignancy; (4) critically missing clinical or imaging data. Ultimately, 157 patients met all criteria and were included in the final analysis. The patient screening process is detailed in Figure 1.

### 2.3 Group definitions

Based on the time interval from endotracheal intubation to tracheostomy, patients were categorized into two groups: the Early Tracheostomy group (ET), defined as undergoing tracheostomy within ≤ 7 days from the initiation of mechanical ventilation via endotracheal intubation; and the Late Tracheostomy group (LT), defined as undergoing tracheostomy after >7 days (13).

The decision and its timing were made at the discretion of the treating clinical team based on a holistic assessment, including factors such as the Glasgow Coma Scale score, intracranial pressure stability, weaning potential, and overall prognosis, acknowledging this as a potential source of confounding.

### 2.4 Data collection

In our study, the term “mechanical ventilation” was specifically defined as invasive positive-pressure ventilation delivered via an endotracheal tube or tracheostomy. This duration threshold was selected to distinguish patients with anticipated prolonged respiratory failure from those receiving only brief peri-procedural ventilation. The ≥48-h timepoint is a common benchmark in critical care research for defining sustained ventilator dependence, and it aligns with the clinical logic that the decision for tracheostomy typically arises after the initial period of stabilization.

Data were independently extracted from the electronic medical record system by two trained researchers using a standardized data collection form, followed by a cross-checking process. The collected data included the following:

#### 2.4.1 Baseline characteristics

Baseline data comprised of demographics (age, gender), relevant comorbidities (hypertension, diabetes mellitus), and admission scores including the Glasgow Coma Scale (GCS) and the Graeb score (calculated from the admission non-contrast head CT). Treatment variables were defined as whether the patient underwent hematoma evacuation and/or external ventricular drainage (EVD).

#### 2.4.2 Imaging metrics

The volumes of parenchymal and intraventricular hematomas were independently measured by two neurosurgeons, blinded to group allocation, using 3D Slicer software (version 4.11; https://slicer.org) on the initial non-contrast head CT scan obtained at admission. An initial segmentation was performed using the “Threshold” tool to select voxels within a Hounsfield unit (HU) range of 50–100, roughly encompassing the hematoma region. This was followed by meticulous manual refinement by the evaluator using the “Paint” and “Erase” tools to ensure that the segmentation boundaries accurately matched the visually identified margins of the hematoma. The procedure was conducted in all three anatomical planes—axial, coronal, and sagittal. A three-dimensional model of the hematoma was then reconstructed, thoroughly inspected, and optimized to ensure anatomical accuracy. The total volume was automatically computed by the software. Two independent evaluators performed the entire process once each, and the average of their results was adopted as the final volume.

#### 2.4.3 Outcome measures

The primary outcome was neurological function at 6 months post-onset, assessed using the modified Rankin Scale (mRS) during outpatient or telephone follow-up. The mRS was prospectively collected as an ordinal variable (scores 0–6). Secondary outcomes included: (1) process measures: total duration of mechanical ventilation and ICU length of stay (LOS); (2) short-term complications assessed until discharge: ventilator-associated pneumonia (VAP), new-onset arrhythmia, shock (defined as requiring continuous vasopressor infusion >4 h to maintain blood pressure), and acute kidney injury (AKI); (3) safety outcomes: in-hospital mortality and tracheostomy-related complications. A composite outcome of “complication-free” status was defined as the absence of any of the specified complications (VAP, new-onset arrhythmia, shock, or AKI) during hospitalization.

Explicit ICU discharge criteria: patients were assessed daily for transfer readiness based on: (1) Respiratory stability: liberation from mechanical ventilation (or stable on a tracheostomy collar with minimal support) for >24 h; (2) Neurological stability: controlled intracranial pressure without intervention for >24 h; and (3) Absence of other active critical issues (e.g., ongoing sepsis).

We define VAP as per the 2016 American Thoracic Society/Infectious Diseases Society of America (ATS/IDSA) guidelines. The diagnosis required all of the following: radiographic: new or progressive infiltrate on chest radiography or CT. Clinical: at least two of the following: fever (>38.0 °C), leukocytosis (>12,000/μl) or leukopenia (< 4,000/μl), or purulent tracheal secretions. Microbiological: significant quantitative culture growth from endotracheal aspirate [≥10^5^ colony-forming units (CFU)/ml] or bronchoalveolar lavage fluid (≥104 CFU/ml), obtained after >48 h of mechanical ventilation. All complications analyzed as outcomes (e.g., VAP, AKI), only cases occurring after the tracheostomy procedure (for both the ET and LT groups) were considered.

### 2.5 Statistical analysis

All statistical analyses were performed using IBM SPSS Statistics (version 26.0) and R language (version 4.2.0). Data normality was assessed using the Shapiro–Wilk test. Continuous variables conforming to a normal distribution are presented as mean ± standard deviation and were compared between groups using the independent samples *t*-test; non-normally distributed data are presented as median (interquartile range) and were compared using the Mann–Whitney *U*-test. Categorical data are expressed as frequency (percentage) and were compared using the Chi-square test or Fisher's exact test, as appropriate. A multivariable logistic regression model was employed to identify independent risk factors for complications. Furthermore, to investigate the association between tracheostomy timing (early vs. late) and the occurrence of short-term complications, a separate multivariable logistic regression analysis was conducted, adjusting for potential confounding factors (such as age, admission GCS score, parenchymal hematoma volume, and intraventricular hemorrhage volume). Variables for adjustment in multivariable logistic regression models were selected *a priori* based on their established clinical prognostic value in intracerebral hemorrhage and an observed association (*P* < 0.1) with the outcome in univariable analysis. The results are reported as adjusted odds ratios (aOR) with their corresponding 95% confidence intervals (CI). All statistical tests were two-sided, and a *P*-value < 0.05 was considered statistically significant.

## 3 Results

### 3.1 Patient baseline characteristics

Baseline characteristics and initial treatments for the entire study cohort (*N* = 157) are presented in Table 1. The Early (ET, *n* = 81) and Late (LT, *n* = 76) groups were well-matched at baseline. There were no statistically significant differences in key demographic factors, including gender distribution, age, or the prevalence of hypertension and diabetes mellitus (all *P* > 0.05). The majority of primary hemorrhages originated in the basal ganglia. Given this limited anatomical distribution and the lack of a statistically significant difference between the two groups. Clinical severity on admission, as measured by the Glasgow Coma Scale score, was identical between groups [median 5 (IQR 4–7)]. Radiological parameters, including the volumes of intraparenchymal and intraventricular hemorrhage, as well as the Graeb scores, were also comparable (all *P* > 0.05). Furthermore, the rates of initial surgical interventions (hematoma evacuation and external ventricular drainage) did not differ significantly between the cohorts. This overall balance supports the validity of subsequent comparative outcomes analysis.

**Table 1:** Baseline characteristics and treatments of the study cohort (*N* = 157).

**Characteristics**	**Early group (ET, *n* = 81)**	**Late group (LT, *n* = 76)**	***P*-value**
**Gender**, ***n*** **(%)**			
M	54 (66.67%)	47 (61.84%)	0.528
F	27 (33.33%)	29 (38.16%)	
Age, mean ± SD	62.43 ± 15.89	61.29 ± 15.56	0.650
Hypertension, *n* (%)	70 (86.42%)	62 (81.58%)	0.407
Diabetes mellitus, *n* (%)	20 (24.69%)	21 (27.63%)	0.675
GCS score on admission, median (IQR)	5 (4, 7)	5 (4, 7)	0.404
Volume of intraparenchymal hemorrhage (ml), mean ± SD	49.96 ± 12.90	47.21 ± 13.95	0.202
Volume of intraventricular hemorrhage (ml), median (IQR)	19.70 (14.60, 24.60)	17.65 (10.98, 24.33)	0.093
Graeb score, median (IQR)	8 (6, 10)	8 (6, 9)	0.155
Hematoma evacuation, *n* (%)	55 (67.90%)	51 (67.11%)	0.915
External ventricular drainage, *n* (%)	66 (81.48%)	59 (77.63%)	0.550

### 3.2 Comparison of clinical outcomes

A comparison of clinical outcomes between the Early Tracheostomy (ET) and Late Tracheostomy (LT) groups is detailed in Table 2. The primary outcome of 6-month functional status (mRS) did not differ significantly between groups (*P* = 0.360). However, the Early Tracheostomy (ET) group demonstrated substantial benefits in key process measures: both the duration of mechanical ventilation [Median Difference (MD) = −6.0 days, *P* < 0.001] and ICU length of stay (MD = −8.0 days, *P* < 0.001) were significantly shorter compared to the Late Tracheostomy (LT) group.

**Table 2:** Comparison of clinical outcomes between groups.

**Outcome measure**	**Early group (ET, *n* = 81)**	**Late group (LT, *n* = 76)**	***P-*value**	**Effect size (95% CI)**
**Primary outcomes**				
6-month mRS score, median (IQR)	4 (3, 5)	4 (3, 5.25)	0.360	
**Process measures**				
Duration of mechanical ventilation (days), median (IQR)	13 (11, 16)	19 (14.75, 22)	< 0.001	MD = −6.0 (−7.2,−4.8)
ICU length of stay (days), median (IQR)	17 (15, 19)	25 (21.75, 28)	< 0.001	MD = −8.0 (−9.5,−6.5)
**Short-term complications**				
**Pulmonary**				
Incidence of VAP, *n* (%)	23 (28.40%)	37 (48.68%)	0.009	OR = 0.42 (0.20, 0.76)
**Cardiovascular**				
New-onset arrhythmia, *n* (%)	15 (18.52%)	25 (32.89%)	0.039	OR = 0.46 (0.22, 0.97)
Shock requiring vasopressors, *n* (%)	20 (24.7%)	31 (40.79%)	0.031	OR = 0.48 (0.24, 0.94)
**Renal**				
Acute Kidney Injury (AKI), *n* (%)	18 (22.22%)	26 (34.21%)	0.095	OR = 0.55 (0.27, 1.11)
**Safety outcomes**				
In-hospital mortality, *n* (%)	15 (18.52%)	17 (22.37%)	0.550	OR = 0.79 (0.36, 1.72)
Tracheotomy-related complications, *n* (%)	5 (6.17%)	7 (9.21%)	0.474	OR = 0.65 (0.20, 2.14)

The incidence of major complications was also lower in the ET group. Patients receiving ET had significantly reduced odds of ventilator-associated pneumonia (OR = 0.42, *P* = 0.009), new-onset arrhythmia (OR = 0.46, *P* = 0.039), and shock requiring vasopressors (OR = 0.48, *P* = 0.031). A non-significant trend toward a lower rate of Acute Kidney Injury was also observed (OR = 0.55, *P* = 0.095). Notably, the two groups were comparable in safety outcomes, with no significant differences in in-hospital mortality or tracheotomy-related complications (all *P* > 0.05).

### 3.3 Multivariable logistic regression analysis

#### 3.3.1 Multivariable logistic regression analysis of independent risk factors

**Figure 2:**
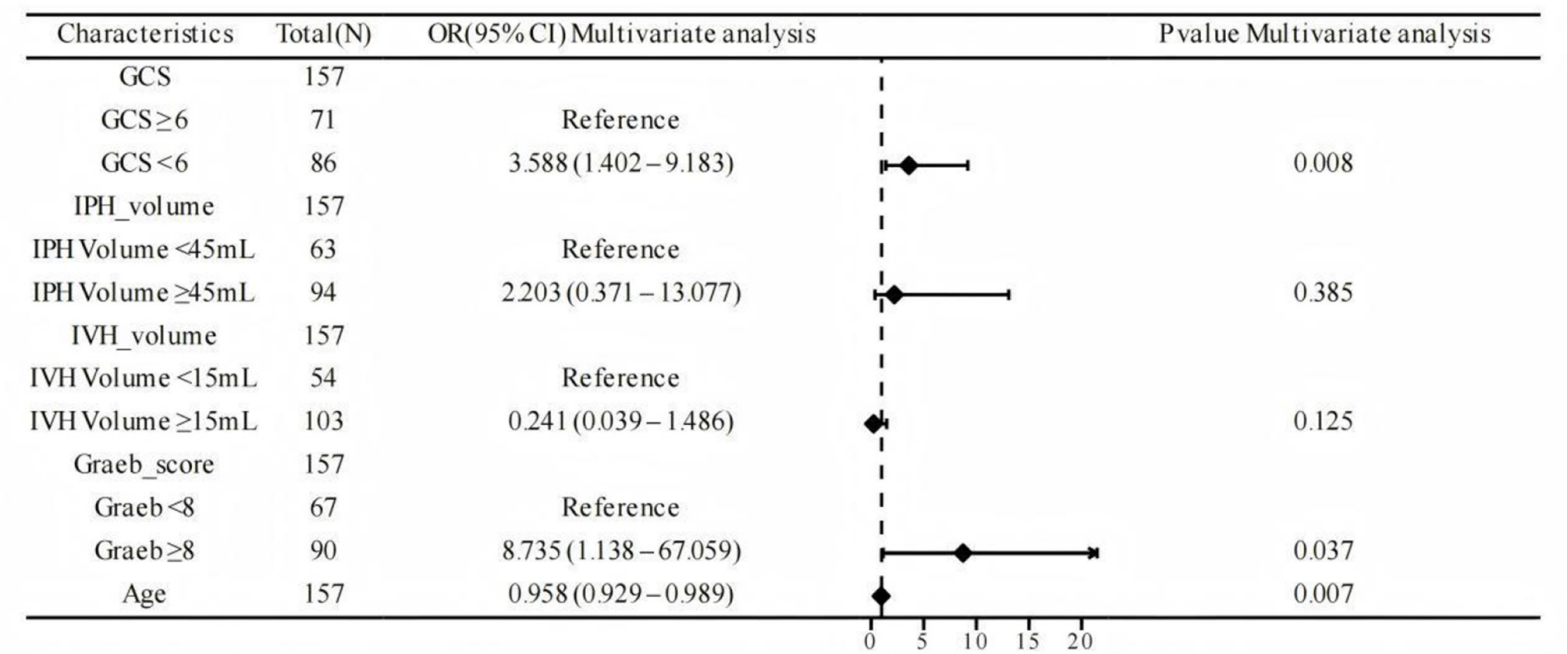
Forest plot of multivariate analysis for independent predictors of the short-term complications. Odds ratios (ORs) with 95% confidence intervals are shown for each variable in the multivariate logistic regression model (*N* = 157). The vertical line at OR = 1 indicates no effect. GCS, Glasgow Coma Scale; IPH, Intraparenchymal Hemorrhage; IVH, Intraventricular Hemorrhage.

Multivariate logistic regression analysis was performed to identify independent risk factors associated with the Short-Term Complications (Figure 2). A Glasgow Coma Scale (GCS) score of < 6 at admission was identified as a significant independent predictor, conferring over a 3.5-fold increased risk (OR 3.588, 95% CI: 1.402–9.183; P = 0.008) compared to patients with a GCS score ≥6. Similarly, a Graeb score of ≥8 was a strong independent risk factor, associated with a markedly elevated odds for the outcome (OR 8.735, 95% CI: 1.138–67.059; P = 0.037). Increasing age was also a significant independent factor. In contrast, the volume of intraparenchymal hemorrhage (IPH; OR 2.203 for ≥45 vs. < 45ml, P = 0.385) and the volume of intraventricular hemorrhage (IVH) (OR 0.241 for ≥15 vs. < 15ml, P = 0.125) were not independently associated with the outcome in the multivariate model.

#### 3.3.2 Association between early tracheostomy and the short-term complications

Multivariable logistic regression identified early tracheostomy as an independent predictor for the Short-Term Complications (Table 3). After adjustment for age, GCS score, Graeb score, and hemorrhage volumes, early tracheostomy was associated with a significantly reduced likelihood of achieving a complication-free course [adjusted Odds Ratio (aOR) = 0.306, 95% CI: 0.114–0.825, P = 0.019]. None of the other covariates included in the model showed a statistically significant association with the outcome.

**Table 3:** Multivariable logistic regression analysis of the association between early tracheostomy and the short-term complications.

**Factor**	**Adjusted odds ratio (aOR)**	**95% confidence interval**	***P*-value**
Early tracheotomy (ref. Late)	0.306	0.114–0.825	0.019
Age (per 1-year increase)	1.029	0.974–1.088	0.305
GCS score (per 1-point increase)	1.281	0.834–1.966	0.258
Graeb score (per 1-point increase)	0.873	0.708–1.077	0.206
IPH volume (per 1-ml increase)	0.949	0.707–1.274	0.727
IVH volume (per 1-ml increase)	1.196	0.490–2.921	0.694

## 4 Discussion

This single-center retrospective cohort study, which incorporated quantitative imaging metrics including parenchymal and intraventricular hematoma volumes for in-depth analysis, demonstrates that in mechanically ventilated patients with severe spontaneous intracerebral hemorrhage extending into the ventricular system, early tracheostomy (ET) did not improve 6-month neurological outcomes despite comparable baseline characteristics, but significantly reduced the duration of mechanical ventilation and ICU length of stay. Moreover, ET was associated with a lower incidence of key short-term complications, including ventilator-associated pneumonia (VAP), new-onset arrhythmia, and shock requiring vasopressor support. Crucially, multivariable analysis identified a GCS score < 6 and a Graeb score ≥8 as independent risk factors for complications, while confirming ET itself as an independent protective factor after adjustment for these confounders.

This study demonstrates significant benefits of early tracheostomy (ET) in key process measures. Compared to the late tracheostomy (LT) group, ET patients had a median reduction of 6 days in mechanical ventilation duration and 8 days in ICU length of stay—a finding consistent with previous studies in mixed critically ill populations (14). These results carry considerable clinical implications, suggesting not only more efficient resource utilization and potential cost reduction but also creating a window for earlier rehabilitation interventions. The shortened ventilation period directly contributed to a lower risk of ventilator-associated pneumonia (VAP). Specifically, the ET group showed a significantly lower VAP incidence than the LT group (28.40 vs. 48.68%). By establishing a stable airway, ET facilitates thorough secretion clearance and oral care while reducing pharyngeal stimulation, thereby directly minimizing the risk of pathogenic microaspiration and substantially mitigating VAP occurrence (15). Moreover, the ET group exhibited significantly lower rates of new-onset arrhythmia and shock requiring vasopressor support, potentially attributable to reduced sedation exposure and improved hemodynamic stability (16).

Importantly, after adjusting for key confounders, multivariable analysis confirmed early tracheostomy as an independent protective factor against short-term complications (aOR = 0.306). These findings suggest that the advantages of ET extend beyond mere “time savings” to exert a positive influence on the patient's overall pathophysiological state. Rather than representing an isolated procedure, early tracheostomy acts as a facilitation that initiates a cascade of favorable clinical responses, ultimately leading to a comprehensive reduction in complication risk (17).

A noteworthy finding was that despite significantly reducing complication risks, early tracheostomy did not translate into improved 6-month neurological function (assessed by the mRS). This highlights the recognized discrepancy between process measures and long-term functional outcomes in clinical research. Our risk model (Figure 2) offers a plausible explanation: a low admission GCS score (< 6) and a high Graeb score (≥8) were identified as powerful independent risk factors for short-term complications. This indicates that the severity of the initial brain injury is the primary driver of ultimate prognosis. Early tracheostomy, as a supportive intervention, effectively mitigates secondary insults (such as VAP and complications from prolonged sedation), thereby optimizing the patient's clinical course in the ICU. However, it is unlikely to reverse the ceiling of neurological recovery predetermined by the severity of the primary injury (18). Consequently, its principal benefits reside in enhancing the quality of care, optimizing resource utilization, and reducing patient suffering, rather than altering the degree of long-term disability (19–22).

This study has several limitations. First, its retrospective, single-center design may be subject to selection bias and residual confounding, despite adjustments using multivariable models. Second, the sample size may have limited the power to detect subtle differences in rare outcomes or specific subgroup analyses. Third, the decision regarding tracheostomy timing was based on clinical judgment rather than randomization, potentially introducing unmeasured confounders (e.g., physician or family preferences). Weaning attempts were part of the standard protocol; however, detailed data on the number or success of attempts prior to tracheostomy were not systematically collected, which is a study limitation. Finally, the study lacked assessments of patient-reported outcomes such as quality of life, as well as economic evaluations such as cost-effectiveness.

Our study provides important evidence for the individualized management of patients with severe ICH. Clinicians should not only focus on those with extremely low initial GCS scores or high Graeb scores but also recognize that for all patients anticipated to require prolonged respiratory support, early tracheostomy represents an effective strategy to proactively improve in-hospital outcomes. It should be regarded not as a last resort after treatment failure, but as an active, preventive component within the critical care pathway. Future research should aim to prospectively validate these findings and further utilize advanced imaging and biomarkers to identify patient subgroups who are not only likely to benefit from process optimization but also retain potential for neurological recovery (23), thereby enabling truly precision-based clinical decision-making (24).

## 5 Conclusion

In conclusion, our study demonstrates that early tracheostomy (within 7 days of mechanical ventilation) in patients with severe intraventricular hemorrhage provides significant clinical benefits by substantially reducing the duration of mechanical ventilation and ICU stay, while effectively lowering the incidence of major pulmonary and cardiovascular complications. The identification of GCS score < 6 and Graeb score ≥8 as independent risk factors, coupled with the establishment of early tracheostomy as an independent protective factor, provides a robust evidence base for individualized clinical decision-making. We recommend that early tracheostomy be considered as a proactive component of the management pathway for patients with severe ICH requiring prolonged mechanical ventilation, particularly those with the identified risk factors. Future prospective studies incorporating advanced neuroimaging biomarkers and patient-centered outcomes are warranted to further refine patient selection and comprehensively evaluate the value of this intervention.

## Data Availability

The raw data supporting the conclusions of this article will be made available by the authors, without undue reservation.
